# Boosting healthy food choices by meal colour variety: results from two experiments and a just-in-time Ecological Momentary Intervention

**DOI:** 10.1186/s12889-019-7306-z

**Published:** 2019-07-22

**Authors:** Laura M. König, Britta Renner

**Affiliations:** Department of Psychology, University of Konstanz, P.O. Box 47, 78457 Konstanz, Germany

**Keywords:** Eating, Colour, Perception, Vegetables, Fruit, Ecological momentary assessment

## Abstract

**Background:**

Dietary guidelines typically specify rather complex goals and indicators for healthy food choices, such as nutrient and energy content patterns. However, translating these complex goals into practice in real life is often a major obstacle for many people. The present studies propose an intervention strategy for boosting healthy food choices by prompting consumers at a meaningful moment with a simple behavioural trigger, that is to eat a colourful lunch. Effectivity and feasibility of this intervention strategy were tested in two laboratory experiments and one real-life, smartphone-based Ecological Momentary Intervention.

**Methods:**

In Studies 1 and 2, 83 / 42 participants self-served four meals (colourful, typical, healthy, and low-calorie) / three meals (colourful, typical, and varied) from a Fake Food Buffet. In Study 3, 80 participants recorded images of 1,210 lunch meals over a period of 3 weeks using mobile visual food recording. In the second week, participants additionally received a daily smartphone prompt to eat a colourful lunch. In all studies, participants were asked to rate the prompts’ feasibility.

**Results:**

Prompting participants to eat a colourful meal increased the proportion of healthy foods consumed compared to typical meals in all three studies. In Studies 1 and 2, colourful meals contained more fruit and vegetables, while in Study 3 the prompt increased vegetable consumption. Furthermore, participants evaluated colourful meals to be the tastiest (Study 1) and most pleasant, and reported that the prompt was easy to follow and act upon.

**Conclusions:**

Results suggest that prompting individuals to eat colourful meals is a promising strategy to facilitate healthy food choices in daily life.

**Trial registration:**

German Clinical Trials Register, DRKS00017552 (Study 3; retrospectively registered on 24^th^ June 2019).

**Electronic supplementary material:**

The online version of this article (10.1186/s12889-019-7306-z) contains supplementary material, which is available to authorized users.

## Background

Despite the wide range of dietary suggestions that are promoted in the media, and the nutritional guidelines provided by scientific societies and federal agencies (including the German Nutrition Society and the U.S. Department of Agriculture) most people do not follow a ‘healthy’ diet (e.g. [1–3]). For example, in Germany, the context of the present set of studies, 87.4 and 59% of people do not consume the recommended amount of vegetables and fruit per day, respectively [4]. Although fruit and vegetable consumption has recently increased in Germany, newer projections suggest that this trend is about to reverse [5].

Nutrient-based dietary guidelines might be difficult to adhere to because interpreting their recommendations into food choices is a complex endeavour (c.f. [6]). Three reasons might account for this difficulty. Firstly, many commonly available dietary guidelines provide a range of information about (1) the amount of energy that should be consumed daily, (2) the amount of foods that should be eaten from each food group, and (3) the ideal ratio of macronutrients consumed per day (e.g. [7]). Ideally, the food consumed within a day fulfils all three criteria, but integrating these different sources of information, however, might not be the dominant decision strategy when making food choices. In fact, one important cue might often be enough to predict food choices [8, 9]. Secondly, nutritional information is sometimes difficult to obtain, e.g. when eating foods that are not packaged and labelled, or when dining in a restaurant. On these occasions, consumers need to infer nutritional information from other cues. However, consumers’ estimations of volume, caloric, and macronutrient content of foods often diverge substantially from actual values [10–13] (König, LM, Ziesemer, K, & Renner, B: Quantifying actual and perceived inaccuracy in estimating the sugar content of foods. Submitted), and can lead to false assumptions about one’s food intake. Thirdly, dietary guidelines have been recently criticised for seldom providing enough information on how to put their recommendations into practice [14], despite previous research showing that this would be necessary for behaviour change to occur [15, 16]. For example, dietary guidelines describe the overall amounts of nutrients and energy that should be consumed within a single day, but they rarely provide concrete information about how a healthy breakfast, lunch and dinner should be complimentarily composed so to ensure that the total variety of foods consumed meets all given criteria [14], further contributing to divergence from the guidelines. While food-based dietary guidelines might be more accessible and instructive to consumers, they are still difficulty to incorporate in everyday life considering the large number of eating-related decisions people encounter daily [17]. As a result, people who intend to change their dietary lifestyle often experience complications in their everyday life due to their diet and report frustration regarding their food choices [18]. Since expected or experienced failure negatively impacts self-efficacy, this might negatively affect both the intention and the perceived ability to adhere to diets [19–21]. New intervention strategies are therefore needed to translate dietary guidelines into practical recommendations that consumers can apply more easily in daily life, making it possible to improve or boost people’s competency in choosing food and facilitating healthy eating [22, 23].

In a similar vein, the Fogg Behaviour Model (FBM) [24, 25] emphasises that the likelihood of changing a behaviour can be increased by reducing its complexity and difficulty. This can be achieved by two means. Firstly, it is suggested to simplify the target behaviour by dividing a demanding behaviour (e.g. healthy eating) into smaller actions that are more easily accomplished (‘tiny habits’ [26]; e.g. eating an apple in the coffee break, or adding a side of vegetables to a lunch). This makes it easier to integrate the changes into daily routines as they require less time, money, cognitive and physical resource to implement, and consequently, perceived ability increases. Most dietary guidelines refer to overall dietary intake, and thus take multiple food groups into account in their recommendations (e.g., [7, 27, 28]). While this is helpful to evaluate overall dietary healthiness, it might provide too many simultaneous starting points for change, e.g. increasing vegetable consumption while decreasing the consumption of meat and sweets. Trying to follow several concurrent recommendations might decrease overall adherence [29], and reduce the sustained effect of a behavioural intervention [30, 31]. An alternative approach, building on the FBM [24], might be to reduce the complexity of the target behaviour by limiting the number of behaviours being targeted, e.g. by reducing the number of targeted food groups. The present set of studies therefore focused on the amount of fruit and vegetables consumed for lunch, the main meal of the day in Germany.

Secondly, according to the FBM, simple triggers should be used as cues to engage in particular actions and highlight when and how a desired behaviour can be performed [24]. Combining these smaller actions and corresponding triggers might allow more effective intervention strategies to be created. In the context of eating, numerous studies have highlighted the importance of visual cues for food choice (e.g. [9, 32–34]). The colour of food might particularly influence what and how much is eaten (e.g. [35]; for reviews, see [36, 37]), suggesting that colour is already a common visual cue for food choice. Moreover, a recent study conducted with German university students suggests that perceived meal colour variety might be related to dietary healthiness, as more colourful meals contained more vegetables and less sweets [38]. Meal colour variety might therefore be a natural visual cue for healthy food choices.

The present set of studies aimed to explore the potential of simple cues for healthy food choices, specifically aiming to test meal colour variety as a new intervention strategy to boost healthy food choices by prompting consumers to compose colourful meals. The first aim was to determine the feasibility and effectiveness of this new intervention strategy in facilitating healthier food choices in a controlled experimental setting, and comparing it to more to common food choice strategies, by using a realistic Fake Food Buffet [39–42]. Specifically, in Study 1, a colourful meal was compared to a typical (e.g. [41, 43]), a healthy (e.g. [40, 42, 44]), and a low calorie lunch meal [45] using a counterbalanced within-subjects design. In Study 2, the colourful meal was compared to a typical and a varied lunch meal (c.f. [27]) as, in the German language, the term colourful may also mean varied. In this vein, the goal was to differentiate whether the effects were actually due to the meal’s colour variety, or were related to choosing a variety of foods. Following the initial studies conducted under controlled conditions, the second aim was to implement and test the intervention strategy in a real-life, smartphone-based Ecological Momentary Intervention [46]. This allowed the effectiveness and feasibility of the intervention strategy to be tested in the participants’ daily lives by evaluating both its effect on food choice and the participants’ perception of the difficulty, complexity, and enjoyment of the intervention. Because the FBM highlights that triggers for a behaviour need to be presented at the right moment to be most effective [24], the intervention was delivered ‘just in time’ [47], i.e. briefly before the participants’ individual lunch times.

## Methods

### Study 1 & 2

Study 1 aimed to compare the feasibility and effectiveness to increase healthy food choices when composing a colourful meal to a typical, a healthy and a low calorie meal. Study 2 aimed to further distinguish the ease and consequences of promoting a colourful meal versus a varied meal. The typical meal was again included to provide a common comparison condition between studies. Both studies were conducted in a controlled experimental setting, using a realistic Fake Food Buffet [39–42].

#### Samples

For Study 1, a power analysis using G*Power 3.1 [48] to detect a small to medium effect (Cohen’s *f* = .15) in a within-subjects design with four measurements yielded an *N* of 62 for 80% power. Eighty-four participants were recruited through the university online study pool. Everyone in the pool was eligible for participation unless they had defective colour vision or had taken part in previous studies with Fake Food buffets. One participant had to be excluded because of a slight impairment of colour vision, reducing the final sample to *N* = 83 (83% female). The sample had a mean (*M*) age of 22.11 (standard deviation (*SD*) = 2.89) and a mean body-mass index (BMI) of 22.15 (*SD* = 3.27, range 16.81–38.77). All participants except one were students representing a range of academic majors including Psychology (64.6%), Politics (8.5%), Linguistics, and Teacher Training Programs (4.9% each). Other academic majors were represented by less than 4% of the sample.

For Study 2, a power analysis using G*Power 3.1 [48] to detect a large effect (Cohen’s *f* = .4; c.f. results Study 1) in a within-design with three measurements yielded an *N* of 12 for 80% power. Forty-two participants (76% female) were recruited using the same procedure as Study 1. The sample had a mean age of 22.21 (*SD* = 6.24) and a mean BMI of 21.54 (*SD* = 2.67, range 17.06–30.19). All participants except one were students, with the majority studying Psychology (81%).

In both studies, participants received 1 h of course credit or 10€ as compensation.

#### Design and procedure

The studies were approved by the University of Konstanz ethics committee and carried out in accordance with the Declaration of Helsinki and the guidelines of the German Psychological Society. Participants were invited to the laboratory for individual sessions and gave written informed consent. Both studies followed a within-subjects design, where participants were initially provided with tableware and asked to serve themselves a meal that they would typically have for lunch from a Fake Food Buffet. When they were finished, they were asked to place the dishes on a serving tray and fill in a short questionnaire. In Study 1, participants were then asked to serve themselves a healthy, a low calorie, and a colourful meal in random order. The buffet was restocked after the second meal. In Study 2, the buffet was restocked immediately after participants self-served the typical meal, and they were then instructed to serve themselves a varied meal and a colourful meal, in random order. Finally, in both studies, participants filled in a questionnaire assessing demographics and evaluations of the choice strategies, while the experimenter unobtrusively weighed and counted the Fake Food items. The participants were then debriefed and paid.

#### Materials and measures

All items used in this study are listed in Additional file 1.

##### Fake food buffet and food choice

**Fig. 1:**
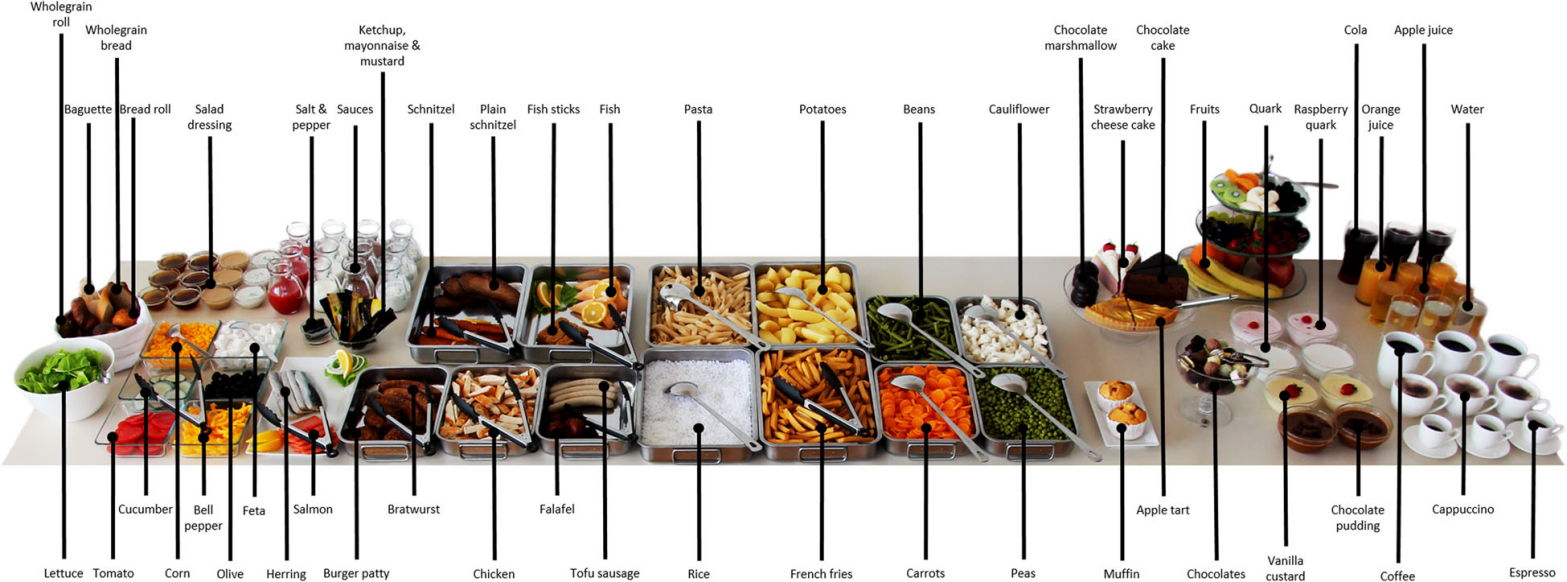
Fake Food Buffet used in Studies 1 and 2

The Fake Food Buffet was derived from Sproesser et al. [42] (see also Bucher et al. [39], Bucher et al. [40], Mötteli et al. [44] for similar buffets), with the addition of vegan falafel and tofu sausages. The buffet included a total of 74 different food items which were placed in serving bowls and arranged on a table to resemble an actual buffet (see Fig. 1). Participants were given a serving tray (55 cm × 35 cm) with a large and a small plate (27 and 21 cm in diameter respectively) and a small bowl (12 cm diameter). The components of the self-served meals were weighed (continuous items, e.g. peas) or counted (e.g. strawberries). The amount of food replicas was converted into the respective amount of real food by multiplying the amount of each replica with a predetermined factor based on a comparison of the replica item and the respective real item (see Sproesser et al. [42]). The foods were grouped into eight categories (vegetables, fruits, grains and starches, protein sources, dairy, fats, sweet extras, and drinks), and standardised to the total weight of the meal according to König and Renner [38].

##### Manipulation check

After each meal, participants were asked to indicate whether they chose foods that were colourful. In Study 1, participants were additionally asked to indicate whether they chose foods that were healthy or low in calories, while in Study 2 they were additionally asked to indicate whether they chose foods that were varied. All items used a six-point Likert scale ranging from (1) “I do not agree at all” to (6) “I totally agree”.

##### Evaluation of the choice strategies

After each meal, participants were asked to indicate how filling the self-served meal would be on a six-point semantic differential from (1) “not at all filling” to (6) “very filling”. After the participants had chosen all meals, they rated the strategies’ feasibility ((1) “very difficult” to (6) “very easy”) and simplicity ((1) “very complex” to (6) “very simple”) and indicated if eating in accordance with the strategy was fun ((1) “not at all fun” to (6) “very fun”) on six-point Likert scales. Furthermore, participants were also asked to rank the choice strategies according to their anticipated taste and feasibility in daily life. A ranking task was used to avoid ceiling effects, as it could be expected that participants generally self-serve tasty meals.

#### Statistical analysis

Data was analysed using IBM SPSS (Version 25). In Study 1, missing values were 1.2% for the evaluation of the healthy and colourful meals due to missing questionnaires and 1.2% for the rankings. Within-subjects Analyses of Variance (ANOVAs) were computed to compare the strategies regarding the overall size, proportion of food groups, and evaluation by the participants. Significant results were followed up by Bonferroni paired comparisons. For all tests, α was set to .05.

### Study 3

This study aimed to implement and test eating colourful meals to facilitate healthy food choices using a smartphone-based Ecological Momentary Intervention.

#### Sample

Sample size estimation in intensive longitudinal studies is difficult when little information about the effects of interest is available [49], so *N* = 108 participants were recruited in accordance with a previous study [38]. Three waves of participants were recruited using an online study pool with each wave containing *n* = 46, *n* = 34, and *n* = 28 participants, respectively. All subjects were eligible for participation unless they had defective colour vision, or had taken part in previous studies assessing perceived meal colour variety. Several participants had to be excluded (1) because they did not complete the study (*n* = 4), (2) because they had difficulties using the study app (*n* = 1), (3) due to data loss because of incorrect settings on the smartphone (*n* = 2), or (4) due to data loss from a server error in the second recruitment wave (*n* = 21).

The final study sample consisted of *N* = 80 participants (88% female) aged from 18 to 43 years (*M* = 22.41, *SD* = 4.00). Their mean BMI was in a normal range (*M* = 22.86, *SD* = 3.52, range 18.04–37.47). There were no differences in age, gender, or BMI across recruitment waves (age: *F*(2,77) = 0.99, *p* = .377; gender: χ^2^(*df* = 2) = 3.40, *p* = .183; BMI: *F*(2,76) = 0.81, *p* = .449). Ninety-nine percent of participants were students: Psychology (51%), Teacher Training Programs with various majors (8%), Law (5%). Other academic majors were represented by less than 5% of the sample. Participants received 2 h of course credit or 20€ as compensation.

In total, *N* = 1,327 meals were logged, but recorded data were incomplete for *n* = 117 meals (e.g. due to missing pictures). Therefore, the present analyses were conducted on *N* = 1,210 unique meals.

#### Design and procedure

The study was carried out in accordance with the Declaration of Helsinki and the guidelines of the German Psychological Society and was approved by the University of Konstanz ethics committee. The study used a single-group within-subjects design. Lunch meals recorded during the first week represent the baseline food consumption. During the second week of the study (intervention period), participants also received a daily prompt reminding them to eat a colourful lunch (“Eat a colourful lunch meal today.”). The time they received the prompt was tailored to the individual by sending it to each participant at the time they stated that they usually bought or prepared their lunch.^1^ During the third week (follow-up), participants again recorded their lunches but without receiving any prompts.

Prior to the study period, participants were invited to the laboratory for individual sessions. They were informed about the study procedure and gave written informed consent. Participants with Android smartphones (*n* = 38) were then asked to install the smartphone application (app) movisensXS (movisens GmbH Karlsruhe; version 0.8.4203; available on Google Play) and download the questionnaires, while participants without an Android smartphone (*n* = 42) received a smartphone (ASUS Padfone Infinity or Motorola Moto G 1st generation) with the app and questionnaires installed. Furthermore, height and weight were measured. The first time they used the app, participants completed a pre-study questionnaire assessing demographic variables and indicated the time of day they usually prepared or went to have their lunch.

**Fig. 2:**
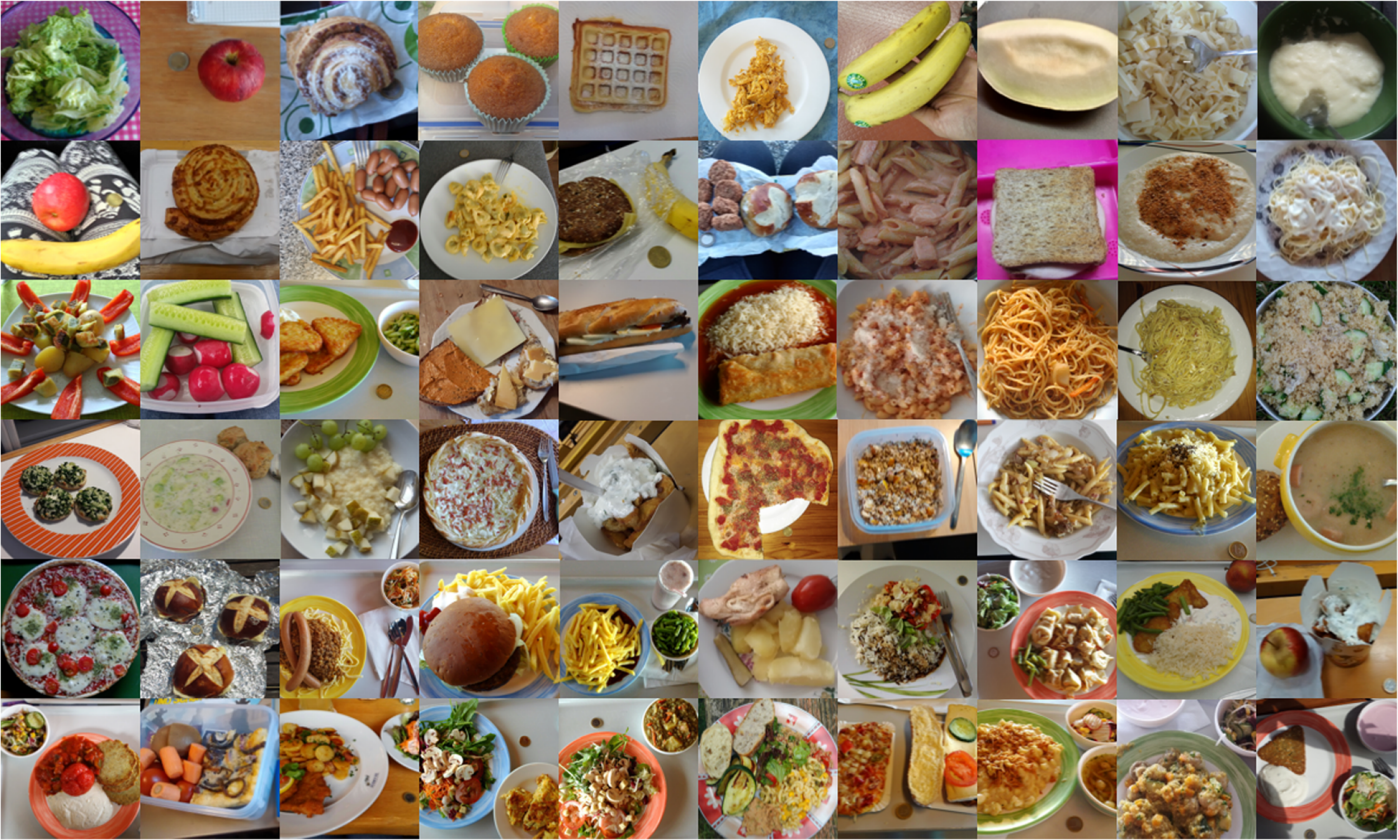
Examples of meal pictures taken by participants in Study 3

The participants were then asked to record their lunch meals in real life for 3 weeks starting the following day by (1) taking a picture (see Fig. 2 for examples), (2) describing the meal, (3) rating the meal’s colours, and (4) taking a picture of any leftovers. Additionally, participants were able to record missing events by indicating (1) that they forgot to record their lunch or (2) that they did not have lunch that day by pressing the relevant button on the app’s home screen (Ziesemer K, König LM, Boushey CJ, Villinger K, Wahl DR, Butscher S, Müller J, Reiterer H, Schupp HT & Renner B: Occurance of and reasons for "missing events" in a mobile dietary assessment: results from three event-based EMA studies. Submitted). Questionnaire data and food pictures were transferred to the server by mobile data or Wi-Fi connections.

After 3 weeks, participants were asked to fill out a post-study questionnaire to evaluate the ease and enjoyment of the prompt. Subsequently, they returned to the laboratory where their weight was measured again, and they were compensated for participating.

#### Materials

All items are listed in Additional file 1.

##### Perceived meal colour variety

Participants rated the meal’s colour on a 100-point visual analogue scale ranging from ‘one colour’ to ‘many colours’ (see also König and Renner [38]).

##### Food intake

Food intake was coded by trained research staff using the participant provided meal descriptions and food pictures following a previously developed coding manual [38] that is based on German dietary guidelines [50]. All foods were assigned to one of seven food groups (vegetables, fruit, grains and starches, animal and other protein sources (i.e. ‘protein’), dairy, fried foods, and desserts and other sugary foods (i.e. ‘sugary extras’)) and their serving sizes were determined based on the pictures taken before and after the meal. As in König and Renner [38], a final food intake score was computed by dividing the serving sizes of all seven categories by the total amount of portions per meal, representing the proportion of the given category in the whole meal.

##### Evaluation of the prompt

After the 3 week study period, the prompt to eat a colourful lunch was evaluated on two 100-point visual analogue scales. Participants indicated whether they found it easy (“Eating colourful meals was easy.”, (0) “I do not at all agree,” (100) “I fully agree”)/ pleasant (“Eating colourful meals is pleasant.”, (0) “I do not at all agree”, (100) “I fully agree”) to eat colourful meals. Participants were also asked to indicate whether they paid attention to the prompts on a 100-point visual analogue scale to assess perceived compliance (“I paid attention to the prompts that I received during the study.” (0) “I do not at all agree”, (100) “I fully agree”).

##### Demographic variables and BMI

When using the app for the first time, participants were asked to indicate their gender, age, current occupation, field of study, and dietary habits. BMI was calculated from measured height and weight. Participants wore light indoor clothing and were asked to remove their shoes before being weighed. Height was measured before the study using a wall-mounted stadiometer, and weight was measured before and after the study using a digital scale (Omron Body Composition Monitor, BF511).

#### Statistical analysis

Following the procedure previously described in König and Renner [38], data was analysed using multilevel linear modelling [51] in R 3.2.3 with the packages lme4 version 1.1–11 [52] and lmerTest 2.0–30 [53]. For all analyses, individual meals defined Level 1, which were nested within participants (Level 2). To analyse the relationships between perceived meal colour variety and intake of the seven food groups, perceived meal colour variety was entered as a Level 1 predictor and thus group-mean centred [54]. Differences in food consumption between baseline, intervention, and follow-up weeks were analysed as a function of time. Models were computed separately to evaluate the difference between baseline and intervention weeks and the difference between baseline and follow-up weeks. Following the procedures suggested by Lischetzke et al. [55], time was dichotomized into (0) baseline and (1) intervention week, and (0) intervention and (1) follow-up week, respectively.

For all analyses, both random slopes and random intercept models were then computed and compared using a deviance test [51]. If the deviance test was significant, differences between participants in the strength and/ or direction of the relationship were assumed and the percentage of positive and negative slopes was computed [51]. For all multilevel models, *quasi-R*^2^ was calculated as an estimate for the effect size, comparing the preferred model to the intercept only model.

Choice strategies were compared using within-subjects ANOVAs with Bonferroni-corrected post-hoc comparisons.

## Results

### Study 1

#### Manipulation check

Within-subjects ANOVAs were conducted to compare the four meals regarding healthiness, energy content, and colourfulness. The participants more strongly agreed that they had chosen healthy foods when putting together the healthy meal (*F*(3, 240) = 46.09, *p* < .001, η_p_^2^ = .37), low calorie foods when putting together the low calorie meal (*F*(3, 243) = 145.13, *p* < .001, η_p_^2^ = .64), and more colourful foods when putting together the colourful meal(*F*(3, 243) = 72.77, *p* < .001, η_p_^2^ = .47). Means and standard deviations are listed in Table 1. The manipulations were therefore successful.

**Table 1:** Means and standard deviations for manipulation check items for studies 1 and 2

Item	Colourful		Typical		Healthy^a^		Low calorie^a^		Varied^b^	
	*M*	*SD*	*M*	*SD*	*M*	*SD*	*M*	*SD*	*M*	*SD*
Study 1: When I put the meal together, I deliberately chose foods that are…										
… healthy.	3.90	1.29	4.09	1.29	5.63	1.04	5.14	1.15		
… low in calories.	2.59	1.21	2.62	1.27	3.84	1.44	5.68	0.89		
… colourful.	5.82	0.65	3.20	1.57	3.78	1.71	3.73	1.71		
Study 2: When I put the meal together, I deliberately chose foods that are…										
… varied.	5.12	1.13	4.55	1.15					5.81	0.51
… colourful.	5.88	0.45	3.55	1.76					4.29	1.60

^a^Was only assessed in Study 1

^b^Was only assessed in Study 2

#### Differences in food consumption

Results are summarised in Table 2. In a first step, total meal weight was compared between conditions, yielding significant differences. Meals in the low calorie condition weighed significantly less than the other meals (*p*s < .001).^2^

**Table 2:** Total weight and proportions of food groups for the choice conditions in studies 1 and 2

	Colourful		Typical		Healthy^a^		Low calorie^a^		Varied^b^		ANOVA			
	*M*	*SD*	*M*	*SD*	*M*	*SD*	*M*	*SD*	*M*	*SD*	*F*	*df*s	*p*	η_p_^b^
Study 1														
Total weight (g)	995.97	311.91	932.58	286.88	917.95	281.90	743.80	267.28			31.81	3, 246	< .001	.28
% vegetables	26.39	9.56	21.31	12.40	29.00	12.22	36.07	15.15			32.66	2.71, 222.00^c^	< .001	.29
% fruit	28.46	14.26	11.65	11.73	20.82	11.88	18.52	14.27			27.59	2.56, 210.22^c^	< .001	.25
% grains and starches	11.61	6.48	12.93	8.73	12.48	8.30	4.86	6.32			24.58	2.64, 216.53^c^	< .001	.23
% protein sources	6.21	6.51	8.53	8.62	8.37	7.97	8.80	10.87			2.57	2.73, 223.57^c^	.061	.03
% dairy	6.45	9.66	5.19	8.60	3.24	6.96	1.28	4.76			8.02	2.55, 209.30^c^	< .001	.09
% fats	10.11	7.76	15.30	10.92	6.42	6.51	4.25	4.64			33.80	2.48, 203.30^c^	< .001	.29
% sugary extras	5.05	9.54	9.26	13.96	0.44	3.03	0.03	0.28			24.26	1.80, 147.53^c^	< .001	.23
% drinks	5.71	10.37	15.83	16.29	19.23	11.73	26.19	12.63			50.34	3, 246	< .001	.38
Study 2														
Total weight (g)	1173.91	770.78	994.57	317.65					1107.28	363.11	1.70	1.23, 50.39^c^	.199	.04
% vegetables	25.21	10.32	22.13	11.59					20.78	10.54	2.65	2, 82	.077	.06
% fruit	29.89	16.92	10.73	11.33					18.51	11.29	28.00	1.73, 70.89^c^	< .001	.41
% grains and starches	11.57	6.83	15.67	9.05					14.01	6.80	4.64	1.59, 65.11^c^	.019	.10
% protein sources	7.19	9.83	7.90	8.60					10.29	9.07	2.34	2, 82	.102	.05
% dairy	4.72	7.39	6.39	9.02					4.52	6.91	.89	2, 82	.415	.02
% fats	9.47	8.31	16.14	10.64					14.50	10.98	7.26	2, 82	.001	.15
% sugary extras	5.36	8.19	5.30	9.94					5.42	8.20	.00	2, 82	.997	.00
% drinks	6.59	9.35	15.74	12.69					11.97	10.50	10.26	2, 82	< .001	.20

^a^Was only assessed in Study 1

^b^Was only assessed in Study 2

^c^Greenhouse-Geisser corrected

In a second step, meals were compared regarding the proportions of food groups. Significant differences between conditions emerged for all food groups except protein sources. Specifically, colourful meals contained more vegetables than typical meals and more fruit than all other meals (*p*s ≤ .002). However, they contained less vegetables than low calorie meals (*p* ≤ .001), and more fats and sweets than healthy and low calorie meals (*p*s ≤ .005). Moreover, they contained more grains and starches and dairy than low calorie meals (*p*s < .001), more dairy than healthy meals (*p* = .021), and less drinks than all other meals (*p*s < .001). Paired comparisons for all food groups are listed in Additional file 2.

#### Evaluation of the choice strategies

Meals differed in the participants’ expectations of satiation (*F*(3, 246) = 53.62, *p* < .001, η_p_^2^ = .40). Low calorie meals were expected to be less filling than the other meals (*p* < .001). Eating low calorie meals was also perceived to be more difficult (*F*(2, 164) = 66.14, *p* < .001, η_p_^2^ = .45), more complex (*F*(2, 164) = 29.73, *p* < .001, η_p_^2^ = .27), and less fun (*F*(2, 164) = 89.20, *p* < .001, η_p_^2^ = .52) than eating healthy or colourful meals (*p*s < .001). Means and standard deviations are listed in Table 3.

**Table 3:** Evaluation of the choice strategies in studies 1 and 2

Criterion	Colourful		Typical^a^		Healthy^b^		Low calorie^b^		Varied^c^	
	*M*	*SD*	*M*	*SD*	*M*	*SD*	*M*	*SD*	*M*	*SD*
Study 1:										
Satiation	5.25	0.97	5.37	0.79	5.10	0.95	4.02	1.13		
Feasibility	4.78	1.09			4.69	1.00	3.36	1.23		
Simplicity	4.64	1.23			4.59	0.98	3.67	1.20		
Fun	4.92	1.10			4.64	1.00	2.98	1.22		
Study 2:										
Satiation	5.45	0.89	5.38	0.76					5.74	0.45
Feasibility	4.69	1.26							4.60	1.11
Simplicity	4.50	1.40							4.29	1.09
Fun	4.81	1.29							4.83	1.19

^a^Was only included for satiation

^b^Was only assessed in Study 1

^c^Was only assessed in Study 2

In the ranking task, healthy meals were ranked first for feasibility by 54.2% of participants, while 37.8% participants ranked colourful meals highest and 8.5% ranked low calorie meals highest. Regarding anticipated taste, colourful meals were ranked highest by 63.4% of participants, while healthy meals were ranked highest by 37.3% and low calorie meals were never ranked first.

### Study 2

#### Manipulation check

Within-subjects ANOVAs were conducted to compare the three meals regarding variety and colourfulness. The participants agreed more strongly to have chosen a variety of foods when putting together the varied meal (*F*(2, 82) = 19.70, *p* < .001, η_p_^2^ = .33), and more colourful foods when compiling the colourful meal (*F*(2, 82) = 45.70, *p* < .001, η_p_^2^ = .53). Means and standard deviations are listed in Table 1. The manipulations were again successful.

#### Differences in food consumption

The results are summarised in Table 2. In a first step, total meal weight was compared between conditions, yielding no significant differences.^3^

In a second step, meals were compared regarding the proportions of food groups. Significant differences were found for fruit, grains and starches, fats, and drinks. Specifically, colourful meals contained a higher proportion of fruit (*p*s ≤ .001) and a lower proportion of fats (*p*s ≤ .025) than the other meals. Moreover, colourful meals contained a smaller proportion of grains and starches than typical meals (*p* = .012), and a smaller proportion of drinks than both typical and varied meals (*p*s ≤ .019). Paired comparisons for all food groups are listed in the Additional file 2.

#### Evaluation of the choice strategies

Meals differed in the participants’ expectations of satiation (*F*(1.75, 71.89) = 5.68, *p* = .007, η_p_^2^ = .12; Greenhouse-Geisser corrected). Participants felt that the typical meal would be less filling than the varied meal (*p* = .001). Colourful and varied meals did not differ in feasibility, simplicity, or fun (*t*s(41) ≥ |1.20|, *p*s ≤ .238). Means and standard deviations are listed in Table 3.

In the ranking task, colourful and varied meals were ranked first for feasibility equally often (50% of participants), while varied meals were ranked first more often regarding anticipated taste and healthiness (taste: 66.7% of participants; healthiness: 71.4% of participants).

### Study 3

#### Relationships between perceived meal colour variety and food intake

**Fig. 3:**
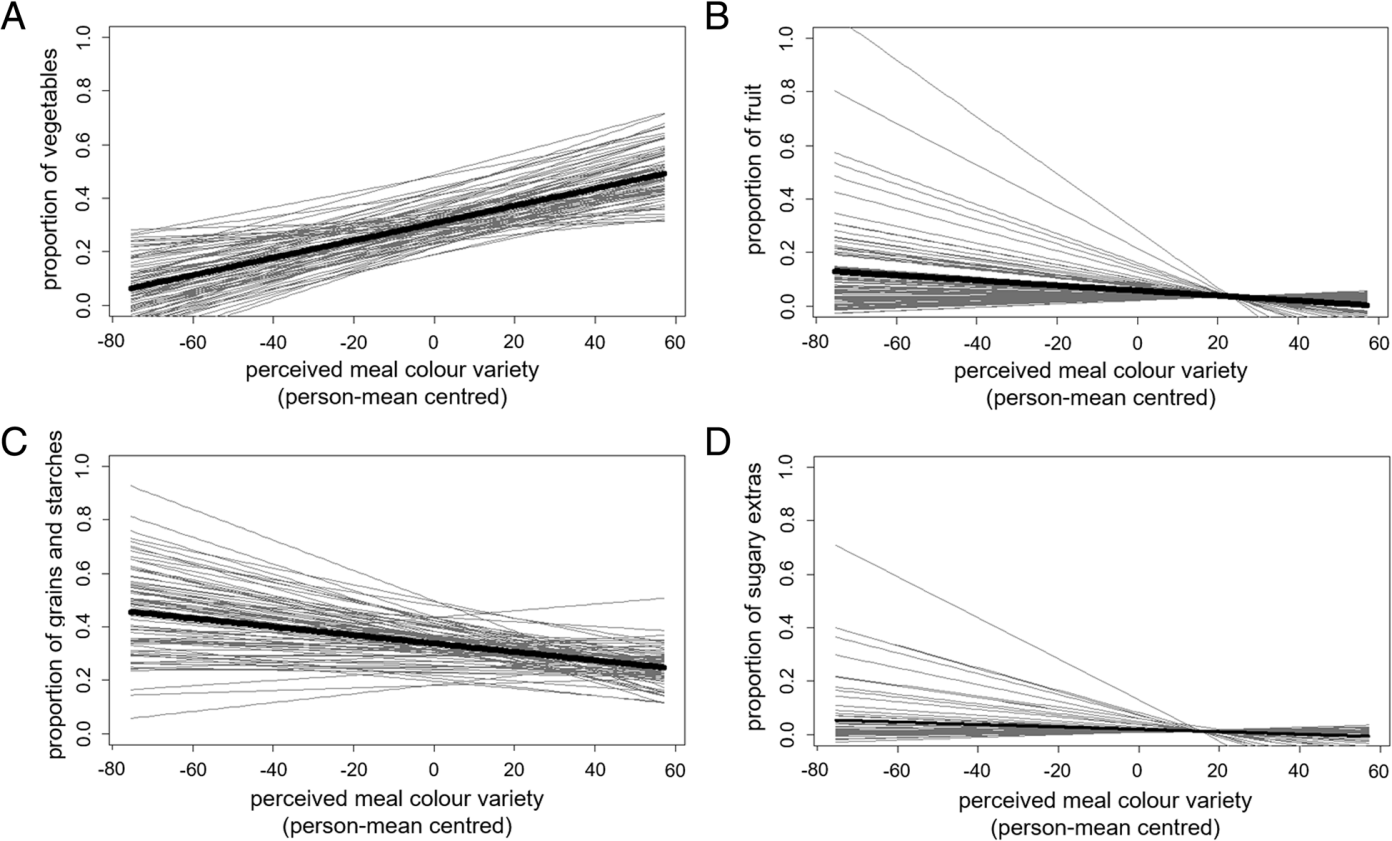
Associations between perceived meal colour variety and proportion of food groups consumed in the meal in Study 3. Each thin grey line represents a regression line for one participant. The thick black line represents the overall regression line. **a** Proportion of vegetables. **b** Proportion of fruit. **c** Proportion of grains and starches. **d** Proportion of sugary extras

Separate multilevel models were computed for all food groups. A significant positive relationship with perceived meal colour variety emerged for vegetables. When comparing the random slopes (*b* = 0.003, *t*(72.79) = 7.73, *p* < .001, *quasi-R*^2^ = .11) and random intercept models (*b* = 0.003, *t*(1132.30) = 9.55, *p* < .001, *quasi-R*^2^ = .07), the random slopes model assuming differences in the individual slopes was preferred (χ^2^(*df* = 2) = 9.82, *p* = .007). The participants therefore differed in the relationship between meal colour variety and proportion of vegetables consumed (see Fig. 3a). Ninety-four percent of slopes were positive, indicating that increased perceived meal colour variety was associated with a higher proportion of vegetables consumed, while 6% of slopes were negative, indicating that for a minority of subjects increased perceived meal colour variety was associated with a lower proportion of vegetables consumed.

A significant negative relationship emerged between perceived meal colour variety and the proportion of fruit consumed. When comparing the random slopes (*b* = −.001, *t*(81.17) = − 2.81, *p* = .006, *quasi-R*^2^ = .10) and random intercept models (*b* = −.001, *t*(1130.00) = − 3.96, *p* < .001, *quasi-R*^2^ = .01), the random slopes model was preferred (χ^2^(*df* = 2) = 66.36, *p* < .001), indicating that the relationship between perceived meal colour variety and the proportion of fruit consumed differed between participants. Sixty-seven percent of slopes were negative, indicating that a greater perceived meal colour variety was associated with a lower proportion of fruit consumed, while 33% of slopes were positive, indicating that a greater perceived meal colour variety was associated with a higher proportion of fruit consumed (see Fig. 3b).

A significant negative relationship also emerged between perceived meal colour variety and the proportion of grains and starches consumed. When comparing the random slopes (*b* = −.002, *t*(65.54) = − 3.87, *p* < .001, *quasi-R*^2^ = .07) and random intercept models (*b* = −.002, *t*(1130.25) = − 5.18, *p* < .001, *quasi-R*^2^ = .02), the random slopes model was preferred (χ^2^(*df* = 2) = 15.03, *p* < .001), indicating differences between participants in the relationship between perceived meal colour variety and the proportion of fruit consumed. Seventy-six percent of slopes were negative, indicating that a greater perceived meal colour variety was associated with a lower proportion of grains and starches consumed, while 34% of slopes were positive, indicating that a greater perceived meal colour variety was associated with a higher proportion of grains and starches consumed (see Fig. 3c).

Lastly, a significant negative relationship emerged between perceived meal colour variety and the proportion of sugary extras consumed. When comparing the random slopes (*b* = −.001, *t*(74.75) = − 2.05, *p* = .044, *quasi-R*^2^ = .11) and random intercept models (*b* = −.000, *t*(1126.25) = − 2.93, *p* = .003,, *quasi-R*^2^ = .01), the random slopes model was preferred (χ^2^(*df* = 2) = 72.37, *p* < .001), indicating that the relationship between perceived meal colour variety and the proportion of sugary extras consumed differed between participants. Sixty-two percent of slopes were negative, indicating that a greater perceived meal colour variety was associated with a lower proportion of sugary extras consumed, while 38% of slopes were positive, indicating that a greater perceived meal colour variety was associated with a higher proportion of sugary extras consumed (see Fig. 3d).

For fried foods, a significant negative relationship emerged for perceived meal colour variety for the random slopes model (*b* = −.000, *t*(141.01) = − 1.98, *p* = .049, *quasi-R*^2^ = .01). However, the deviance test (χ^2^(*df* = 2) = 4.21, *p* = .122) comparing the random slopes to the random intercept model preferred the random intercept model (*b* = −.000, *t*(1123.69) = −-1.76, *p* = .079, *quasi-R*^2^ = .00), which did not reach significance.

No significant relationships with perceived meal colour variety were found for protein and dairy (see Table 4 for a summary of all models).

**Table 4:** Results of the multilevel models to analyse the relationship between perceived meal colour variety and the consumption of seven food groups

Predictor	Random slopes model (fixed effects)					Random intercept model (fixed effects)				
	*b*	*SE*	*t*	*df*	*p*	*b*	*SE*	*t*	*df*	*p*
Model 1: proportion of vegetables										
Intercept	0.307	0.011	27.67	80.33	< .001	0.307	0.011	27.66	80.40	< .001
Perceived meal colour variety	0.003	0.000	7.73	72.79	< .001	0.003	0.000	9.55	1132.30	< .001
Model 2: proportion of fruit										
Intercept	0.057	0.007	7.86	79.83	< .001	0.057	0.007	8.02	77.53	< .001
Perceived meal colour variety	−0.001	0.000	−2.81	81.17	.006	−.001	0.000	−3.96	1130.00	< .001
Model 3: proportion of grains and starches										
Intercept	0.338	0.011	29.96	78.71	< .001	0.338	0.011	29.98	78.76	< .001
Perceived meal colour variety	−0.002	0.000	−3.87	65.54	< .001	−.002	0.000	−5.18	1130.25	< .001
Model 4: proportion of sugary extras										
Intercept	0.019	0.004	4.83	69.97	< .001	0.018	0.004	4.91	72.82	< .001
Perceived meal colour variety	−0.001	0.000	−2.05	74.45	.044	−0.000	0.000	−2.93	1126.25	.003
Model 5: proportion of protein										
Intercept	0.110	0.010	10.80	81.00	< .001	0.110	0.010	10.79	81.02	< .001
Perceived meal colour variety	0.000	0.000	1.09	68.75	.278	0.000	0.000	1.16	1131.90	.248
Model 6: proportion of dairy										
Intercept	0.115	0.009	13.45	80.27	< .001	0.115	0.009	13.45	80.20	< .001
Perceived meal colour variety	−0.000	0.000	−0.87	556.23	.387	−0.000	0.000	−0.83	1131.74	.409
Model 7: proportion of fried foods										
Intercept	0.054	0.006	8.93	73.19	< .001	0.054	0.006	9.00	71.67	< .001
Perceived meal colour variety	−0.000	0.000	−1.98	141.01	.049	−0.000	0.000	−1.76	1123.69	.079

#### Impact of the prompt to eat a colourful lunch on food consumption

##### Differences between baseline and intervention weeks

A significant difference between the baseline and intervention weeks emerged for vegetables consumed. When comparing the random slopes (*b* = 0.04, *t* (548.83) = 2.16, *p* = .031, *quasi-R*^2^ = .02) and random intercept models (*b* = 0.04, *t* (768.21) = 2.20, *p* = .028, *quasi-R*^2^ = .02), the random intercept model assuming no differences in the individual slopes was preferred (χ^2^(*df* = 2) = 0.69, *p* = .709). Thus, the difference between baseline and intervention weeks regarding the proportion of vegetables consumed was comparable between participants. Results indicate that the participants consumed a greater proportion of vegetables during the intervention week compared to the baseline week.

A significant difference between baseline and intervention weeks emerged for dairy consumption. When comparing the random slopes (*b* = − 0.04, *t*(81.50) = − 3.16, *p* = .002, *quasi-R*^2^ = .02) and random intercept models (*b* = − 0.04, *t* (766.80) = − 3.17, *p* = .002, *quasi-R*^2^ = .02), the random intercept model assuming no differences in the individual slopes was preferred (χ^2^(*df* = 2) = 0.79, *p* = .675). Thus, the difference between baseline and intervention weeks regarding the proportion of dairy consumed was comparable between participants. Results indicate that the participants consumed a smaller proportion of dairy products during the intervention week compared to the baseline week.

For all other food groups, no significant differences emerged between the baseline and intervention weeks (see Table 5).

**Table 5:** Results of the multilevel models to compare differences in food consumption between baseline and intervention weeks

Predictor	Random slopes model (fixed effects)					Random intercept model (fixed effects)				
	*b*	*SE*	*t*	*df*	*p*	*b*	*SE*	*t*	*df*	*p*
Model 1: proportion of vegetables										
Intercept	0.287	0.015	19.34	89.81	< .001	0.287	0.016	18.55	150.42	< .001
Time^1^	0.037	0.017	2.16	548.83	.031	0.037	0.017	2.20	768.21	.028
Model 2: proportion of fruit										
Intercept	0.056	0.008	6.63	154.57	< .001	0.056	0.009	5.97	173.98	< .001
Time^1^	0.003	0.012	0.27	152.02	.791	0.003	0.011	0.23	769.56	.821
Model 3: proportion of grains and starches										
Intercept	0.339	0.014	24.56	97.60	< .001	0.339	0.015	22.61	141.70	< .001
Time^1^	0.005	0.016	0.32	333.90	.747	0.006	0.016	0.35	766.16	.726
Model 4: proportion of sugary extras										
Intercept	0.018	0.005	3.56	63.44	< .001	0.018	0.005	3.88	245.96	< .001
Time^1^	−0.002	0.007	−0.22	113.23	.830	−0.001	0.006	−0.19	780.05	.849
Model 5: proportion of protein										
Intercept	0.109	0.012	9.02	81.67	< .001	0.109	0.012	8.94	133.04	< .001
Time^1^	0.001	0.012	0.09	746.70	.927	0.001	0.012	0.10	765.00	.923
Model 6: proportion of dairy										
Intercept	0.132	0.011	12.25	79.87	< .001	0.132	0.010	12.83	152.88	< .001
Time^1^	−0.037	0.012	−3.16	81.51	.002	−0.037	0.012	−3.17	766.80	.002
Model 7: proportion of fried foods										
Intercept	0.058	0.008	7.07	74.34	< .001	0.057	0.008	7.23	160.83	< .001
Time^1^	−0.009	0.010	−0.92	75.21	.361	−0.009	0.009	−0.91	766.12	.365

Time: 0 = baseline week, 1 = intervention week

##### Differences between baseline and follow-up week

Between baseline and follow-up weeks, no significant differences were found (*b*s *≤* |0.02|, *t*s(≥ 74.40) *≤* |1.31|, *p*s ≥ .190), indicating that food consumption during the follow-up week returned to the baseline level when prompts were no longer sent.

#### Evaluation of the prompt

Participants indicated that they found eating colourfully is something that is rather easy for them to do (*M* = 57.96, *SD* = 24.87). They also indicated that eating colourfully is pleasant (*M* = 70.79, *SD* = 27.95), and self-rated compliance was satisfactory (*M* = 60.36, *SD* = 26.89).

## General discussion

The present set of studies aimed to test meal colour variety as a new intervention strategy to boost healthy food choices by prompting consumers to choose colourful meals. The strategy was tested in two laboratory experiments using a Fake Food Buffet, and in a real-life, smartphone-based Ecological Momentary Intervention. In all studies, both the effect of this strategy on meal composition and its feasibility were evaluated.

In Studies 1 and 2, the proportions of the eight food groups presented in colourful meals were compared to those found in typical, healthy, low calorie, and varied meals. Colourful meals contained a greater proportion of healthy foods such as fruit and vegetables, and a smaller proportion of unhealthy foods such as fats and oils. Although the difference in self-serving vegetables did not reach significance in Study 2, the effect points in the same direction (25.21% vs 22.13%) and still constitutes a small effect (*d* = 0.28, [56]). Thus, encouraging colourful meals has the potential to increase dietary healthiness compared to the meals in a typical diet. Moreover, the composition of colourful meals was healthier than the composition of varied meals, indicating that the specific instruction to compiling colourful meals goes beyond the effect of encouraging variety, which is currently included in dietary guidelines such as those of the German Nutrition Society [27]. Eating a colourful meal seems to specifically increase the proportion of healthy foods and decrease the proportion of fats and oils in the meal.

Colourful meals contained a larger proportion of fruit than healthy and low calorie meals, but also a somewhat higher proportion of sweets and fats. This was also mirrored in an increased calorie content of colourful meals compared to healthy and low calorie meals. Interestingly, participants expected low calorie meals to be less filling than colourful meals, which mirrors the smaller meal size. Although low calorie meals had a somewhat more favourable meal composition, mainly due to containing less unhealthy foods, decreased satiation and perceived healthiness suggest that eating low calorie meals might not decrease overall food intake, or might even lead to increased food intake (e.g. [57, 58]). However, future studies are needed to better understand the impact of the different food choice strategies on overall dietary composition and nutrient intake across multiple meals.

Study 3 investigated whether a prompt to eat a colourful lunch elicited beneficial changes in real-life food consumption. When prompted to eat a colourful lunch meal, participants consumed a larger proportion of vegetables. Changes in vegetable consumption between the baseline and intervention weeks were comparable between participants, suggesting that prompting to eat a colourful lunch might be a generic approach to facilitating healthy eating. At the same time, when prompted to consume colourful meals, participants consumed a smaller proportion of dairy, suggesting a specific compensation of reducing dairy in order to increase the amount of vegetables. Although the specific consumption of dairy itself was not related to perceived meal colour variety in the present and a previous study [38], it might have been substituted because of its mainly white colour. Consumption of other food groups was unaffected by the prompt. For fruit and sweet extras, this could be because these two categories might not typically be considered as a core component of a lunch meal. On the other hand, grains and starches, despite their similar colouring, are usually readily available in most settings and may also be considered a satiating meal component that is not suitable for substitution.

The differences observed in consumption between the baseline and intervention weeks are small yet meaningful, considering that the present study tested the effectiveness of prompting as one single behaviour change technique (BCT), compared to only self-monitoring of food intake in the baseline and follow-up weeks. Most online and web-based dietary interventions combine multiple BCTs (e.g. [59], see also [60]). For example, a prompt to ‘eat your colours’ (p. 34) was used among other prompts in a text-messaging intervention and additionally paired with health information [61]. Similarly, challenges to eat vegetables of a certain colour were used in a gamified app to prompt vegetable consumption, again amongst other challenges and BCTs. Interestingly, although this app included multiple BCTs and gamified challenges, the effects of this app-based intervention and the intervention presented here were of similar magnitude [62, 63]. Nonetheless, future studies should investigate whether the efficacy of the presented intervention could be increased, e.g. by combining the prompt with other BCTs, such as goal setting or feedback [64, 65].

After the intervention week, vegetable and dairy consumption returned to baseline levels as 1 week is not long enough to form a new habit. The literature suggests that this might take at least 14 [66] and up to 254 days [67], with longer intervention periods potentially further increasing automaticity [66, 68]. While the present study provides first evidence that prompting consumers to eat colourful meals induces behaviour change, future studies are needed to test whether immediate changes in vegetable consumption can translate into long-term behaviour change.

The literature also suggests that eating colourful meals might lead to increased consumption [32]. In Studies 1 and 2, the weight of the colourful meal was comparable to the weight of the typical, healthy, and varied meals. In Study 3, when prompted to eat colourful meals, participants consumed a larger proportion of vegetables but a smaller proportion of dairy, suggesting a specific compensation. The present set of studies therefore do not support the notion that increasing meal colour variety leads to increased food consumption, but instead suggest that eating colourful meals might lead to a shift in meal composition that increases dietary healthiness without impacting the overall amount consumed.

Comparing the three presented studies further highlights the importance of taking the environment into account when designing and evaluating food choice strategies (c.f., [69–71]). In Studies 1 and 2, colourful meals contained the highest proportion of fruit, while in Study 3, meal colour variety was negatively related to the proportion of fruit consumed (see also [38]), and the prompt to eat a colourful meal did not affect fruit consumption. This might be due to the different study settings. The selection of whole and cut fruit offered on the Fake Food Buffet that was used in Studies 1 and 2 might have increased consumption across choice conditions [40, 41]. In Study 3, on the other hand, consumption of fruit was generally very low. Two factors might account for this. First, the university canteen, in which many of the study participants might have occasionally consumed lunch during the study period, only offers a limited selection of fruit. Second, having a large selection of fruit available at home might not be feasible for some participants, especially those in single households. These results suggest that the effectivity of eating colourful meals might be impacted by the availability of fruit and vegetables in the food choice situation (c.f. [72, 73]). As interventions on the individual and on the structural level might interact [74], future research should further explore in which environments it is especially helpful to prompt consumers to eat colourful meals, generating insights on when and where using the strategy is most effective.

In addition to studying changes in behaviour, the present studies also evaluated the feasibility of eating colourful meals. Pronounced differences emerged in Study 1 between colourful and low calorie meals. Low calorie meals were consistently ranked as more difficult to put together than colourful meals, which is in line with the FBM’s supposition that reducing the complexity of a behaviour, for instance by replacing numeric values that need to be derived from knowledge with easily-accessible visual representations, should lead to an increase in the perceived ability [24]. Colourful meals were also ranked as tastier than both low calorie and healthy meals. Since both liking and convenience are important motives for food choice, and liking in particular was ranked higher than health and weight control motives in large-scale and cross-cultural surveys [75, 76], this finding further supports the notion that eating colourful meals might be an effective and feasible strategy to facilitate healthy eating.

These findings were further supported and extended by Study 3, which showed that prompting consumers to eat a colourful meal was also feasible in daily life. Participants indicated that eating colourful meals was both easy and pleasant, and self-rated compliance to the prompt was satisfactory. Previous qualitative studies support this result, as colourful meals have been shown to stimulate the consumer’s senses and enhance meal satisfaction [77, 78], and preparing colourful meals has previously been identified as an intuitive strategy for caregivers to provide children with nutritious food [79].

Interestingly, although participants rated eating healthy meals and eating colourful meals to be equally complex and difficult, eating healthy meals was perceived to be more feasible than eating colourful meals in Study 1. This might reflect that participants might be more familiar with eating healthy meals than with eating colourful meals. Future studies need to test this assumption and could also explore how perceived feasibility changes due to an intervention that promotes eating colourful meals.

Prompting participants “just in time” to eat a colourful meal might have further contributed to the feasibility and effectiveness of the intervention tested in Study 3, as it reduces the effort needed to remember the strategy and apply it in a meaningful moment, i.e. when support is needed [80]. While in the present study a single time was set for all prompts that each participant received, future studies should use algorithms to adaptively determine meaningful moments and so increase the fit between prompt and situation by dynamically responding to people’s immediate situations and needs [81]. In addition, future studies might need to test the generalizability of the prompt across the day, e.g. by prompting participants to eat colourfully once a day instead of before every meal, to potentially reduce disturbing interruptions of ongoing activities [82]. Finally, future studies might also test effectivity in a randomized intervention within participants as it is increasingly used in N-of-1 research [83] to allow for testing of potential carry-over effects between days.

While the present set of studies offers a promising strategy to facilitate healthy food choices, some limitations need to be acknowledged. The present studies focused on lunch meals reported by western students. Although this strategy could be effective for dinner as both meals usually comprise the same items, breakfasts and snacks might incorporate colourful but unhealthy items, such as breakfast cereals or wine gums. Future studies therefore need to test the generalisability of the findings to other meal types. Furthermore, generalisation to other cultures and age groups should be tested, as they might, for instance, differ in colour perception [84].

## Conclusions

Building on the FBM, the present set of studies develops a new strategy for healthy eating by identifying fruit and vegetable consumption as a target behaviour and showing meal colour variety to be a simple, effective, and feasible cue for action in two laboratory studies and an Ecological Momentary Intervention. This set of studies provides a promising foundation for future (mobile) health promotion programmes in which eating a colourful meal could be communicated to consumers to boost their food choice competences.

## Additional files

Additional file 1 : Items used in Studies 1, 2 and 3. All items used in Studies 1, 2 and 3, including sources. (DOCX 23 kb)

Additional file 2 : Additional results for Studies 1 and 2. Results of paired comparisons (Bonferroni corrected) for differences in food consumption for Studies 1 and 2. (DOCX 19 kb)

## Data Availability

The datasets analysed during the current set of studies are available from the corresponding author on reasonable request.

## Abbreviations

ANOVA: Analysis of Variance
BCT: Behaviour change technique
BMI: Body Mass Index
FBM: Fogg Behaviour Model
M: Mean
SD: Standard deviation

## Footnotes

1 Triggers were set between 7:00 am and 4:00 pm, median = 12:00 am.

2 When comparing the total amount of calories between conditions, significant differences emerged (*F*(2.72, 222.98) = 85.02, *p* < .001, *partial* η^2^ = .51, Greenhouse-Geisser corrected ɛ = .91). All conditions differed significantly from each other (*p*s ≤ .001) with the exception of typical and colourful meals (*p* = 1.000; *M*_typical_ = 819.60, *SD*_typical_ = 337.48; *M*_colourful_ = 836.45, *SD*_colourful_ = 373.26; *M*_healthy_ = 578.61, *SD*_healthy_ = 305.71; *M*_low calorie_ = 363.90, *SD*_low calorie_ = 174.84).

3 When comparing the total amount of calories between conditions, significant differences emerged (*F*(2, 82) = 4.73, *p* = .011, *partial* η^2^ = .10). Varied meals contained more calories than typical meals (*p* = .015, *M*_typical_ = 818.01, *SD*_typical_ = 320.34; *M*_varied_ = 1030.04, *SD*_varied_ = 523.80). All other comparisons did not reach significance (*p*s ≥ .117).
