# Supplementary material for: Boosting healthy food choices by meal colour variety: results from two experiments and a just-in-time Ecological Momentary Intervention
Source: BMC Public Health. 2019 Jul 22;19:975. doi: 10.1186/s12889-019-7306-z (PMC6647103; doi:10.1186/s12889-019-7306-z)
Supplement: Supplementary file 1 — : Items used in Studies 1, 2 and 3. All items used in Studies 1, 2 and 3, including sources. (DOCX 23 kb) [file 12889_2019_7306_MOESM1_ESM.docx]

# Questionnaire Studies 1 and 2

## Questionnaire after every buffet

| **Item** | **Response format** | **Used in Study…** |
| --- | --- | --- |
| When composing the meal, I deliberately chose foods that are low in calories. | (1) I do not agree at all to (6) I totally agree | 1 |
| When composing the meal, I deliberately chose foods that are healthy. | (1) I do not agree at all to (6) I totally agree | 1 |
| When composing the meal, I deliberately chose foods that are colourful. | (1) I do not agree at all to (6) I totally agree | 1, 2 |
| When composing the meal, I deliberately chose foods that are varied. | (1) I do not agree at all to (6) I totally agree | 2 |
| The chosen meal is… | (1) not at all filling to (6) very filling | 1, 2 |

*Note.* These items were developed for this set of studies.

## Post-study questionnaire

| **Item** | **Response format** | **Used in Study…** | **Source** |
| --- | --- | --- | --- |
| Your age: | [open text field] | 1, 2 | König and Renner (2018) |
| Your gender: | (1) female  (2) male | 1, 2 | König and Renner (2018) |
| Are you currently… | (1) employed  (2) a student  (3) without employment  (4) retired  (5) housewife/ houseman | 1, 2 | König and Renner (2018) |
| If you are a student, please indicate your major: | [open text field] | 1, 2 | König and Renner (2018) |
| Do you suffer from colour blindness (e.g. red-green-blindness)? | (1) yes  (2) no | 1, 2 |  |
| Do you suffer from any other visual impairment that may impact your colour vision? | (1) yes  (2) no | 1, 2 |  |
| How tall are you? | I am [open text field] meters tall. | 1, 2 |  |
| How much do you weigh? | I weigh [open text field] kg. | 1, 2 |  |
| Do you eat according to specific dietary principles? (multiple answers allowed) | - No - Yes, calorie restriction for weight loss - Yes, to treat an illness (e.g. diabetes, allergies, metabolic disorder, gout) - Yes, vegetarian - Yes, vegan, - Yes other [+ open text field] | 1, 2 | König and Renner (2018) |
| Eating healthy meals is… | (1) very difficult to (6) very easy  (1) very complex to (6) very simple  (1) not at all fun to (6) very fun | 1 |  |
| Eating meals low in calories is… | (1) very difficult to (6) very easy  (1) very complex to (6) very simple  (1) not at all fun to (6) very fun | 1 |  |
| Eating colourful meals is… | (1) very difficult to (6) very easy  (1) very complex to (6) very simple  (1) not at all fun to (6) very fun | 1, 2 |  |
| Eating varied meals is… | (1) very difficult to (6) very easy  (1) very complex to (6) very simple  (1) not at all fun to (6) very fun | 2 |  |
| Which prompt (healthy, low calorie or colourful) can be applied easiest to daily life?  Please rank the three options accordingly. | Options:   1. Healthy 2. Low calorie 3. Colourful | 1 |  |
| Which prompt (healthy, low calorie or colourful) leads to the tastiest meals?  Please rank the three options accordingly. | Options:   1. Healthy 2. Low calorie 3. Colourful | 1 |  |
| Which prompt (varied or colourful) can be applied easiest to daily life?  Please rank the two options accordingly. | Options:   1. Varied 2. Colourful | 2 |  |
| Which prompt (varied or colourful) leads to the tastiest meals?  Please rank the three options accordingly. | Options:   1. Varied 2. Colourful | 2 |  |

# Questionnaires Study 3

## Pre-study questionnaire

| **Item** | **Response format** | **Source** |
| --- | --- | --- |
| Your gender: | (0) female  (1) male | König and Renner (2018) |
| Your age: | [open text field] | König and Renner (2018) |
| Are you currently… | (1) employed  (2) a student  (3) without employment  (4) retired  (5) housewife/ houseman | König and Renner (2018) |
| If you are a student, please indicate your major: | [open text field] | König and Renner (2018) |
| Do you suffer from colour blindness (e.g. red-green-blindness)? | (0) no  (1) yes |  |
| Do you suffer from any other visual impairment that may impact your colour vision? | (0) no  (1) yes |  |
| Do you eat according to specific dietary principles? (multiple answers allowed) | - No - Yes, calorie restriction for weight loss - Yes, to treat an illness (e.g. diabetes, allergies, metabolic disorder, gout) - Yes, vegetarian - Yes, vegan, - Yes other [+ open text field] | König and Renner (2018) |
| At what time do you usually start to prepare lunch? | [set timer] |  |

## Recording lunch meals

| **Item** | **Response format** | **Source** |
| --- | --- | --- |
| Please take a picture of your meal. | [camera app] | König and Renner (2018) |
| Please provide a please description of your meal. | [open text field] | König and Renner (2018) |
| Please rate the meal’s colour variety.  My meal contains… | (0) one colour to (100) many colours | König and Renner (2018) |
| Did you clear your plate? | (1) yes  (2) no |  |
| (If plate was not cleared)  Please take a picture of the leftovers. | [camera app] |  |

## Post-study questionnaire

| **Item** | **Response format** |
| --- | --- |
| I paid attention to the prompts that I received during the study. | (0) I do not at all agree to (100) I fully agree |
| Eating colourful meals was easy. | (0) I do not at all agree to (100) I fully agree |
| Eating colourful meals is pleasant. | (0) I do not at all agree to (100) I fully agree |

# References

König, L. M., & Renner, B. (2018). Colourful = healthy? Exploring meal colour variety and its relation to food consumption. *Food Quality and Preference, 64*, 66-71. doi: 10.1016/j.foodqual.2017.10.011
