# Supplementary material for: Boosting healthy food choices by meal colour variety: results from two experiments and a just-in-time Ecological Momentary Intervention
Source: BMC Public Health. 2019 Jul 22;19:975. doi: 10.1186/s12889-019-7306-z (PMC6647103; doi:10.1186/s12889-019-7306-z)
Supplement: Supplementary file 2 — : Additional results for Studies 1 and 2. Results of paired comparisons (Bonferroni corrected) for differences in food consumption for Studies 1 and 2. (DOCX 19 kb) [file 12889_2019_7306_MOESM2_ESM.docx]

Table S1. Results of paired comparisons (Bonferroni corrected) for differences in food consumption, Study 1.

|  |  | % vegetables | % fruit | % grains and starches | % protein sources | % dairy | % fats | % sugary extras | % drinks |
| --- | --- | --- | --- | --- | --- | --- | --- | --- | --- |
|  |  | *p* | *p* | *p* | *p* | *p* | *p* | *p* | *p* |
| colourful | typical | .001 | < .001 | 1.000 | .128 | 1.000 | .002 | .067 | < .001 |
|  | healthy | .278 | .002 | 1.000 | .169 | .021 | .005 | < .001 | < .001 |
|  | low calorie | < .001 | < .001 | < .001 | .194 | < .001 | < .001 | < .001 | < .001 |
| typical | healthy | < .001 | < .001 | 1.000 | 1.000 | .415 | < .001 | < .001 | .474 |
|  | low calorie | < .001 | .006 | < .001 | 1.000 | .004 | < .001 | < .001 | < .001 |
| healthy | low calorie | < .001 | .933 | < .001 | 1.000 | .191 | .061 | 1.000 | < .001 |

|  |  | % vegetables | % fruit | % grains and starches | % protein sources | % dairy | % fats | % sugary extras | % drinks |
| --- | --- | --- | --- | --- | --- | --- | --- | --- | --- |
|  |  | *p* | *p* | *p* | *p* | *p* | *p* | *p* | *p* |
| colourful | typical | .454 | < .001 | .012 | 1.000 | .789 | .001 | 1.000 | < .001 |
|  | varied | .057 | .001 | .062 | .068 | 1.000 | .025 | 1.000 | .019 |
| typical | varied | 1.000 | .001 | .945 | .345 | .815 | 1.000 | 1.000 | .230 |

Table S2. Results of paired comparisons (Bonferroni corrected) for differences in food consumption, Study 2.
